# The Potential Benefit of Complementary/Alternative Medicine in Cardiovascular Diseases

**DOI:** 10.1155/2012/125029

**Published:** 2012-06-18

**Authors:** Ke-ji Chen, Ka Kit Hui, Myeong Soo Lee, Hao Xu

**Affiliations:** ^1^Cardiovascular Center, Xiyuan Hospital, China Academy of Chinese Medical Sciences, Beijing 100091, China; ^2^Center for East-West Medicine, University of California at Los Angeles, Santa Monica, CA 90404, USA; ^3^Medical Research Division, Korea Institute of Oriental Medicine, Daejeon 305-811, Republic of Korea

Cardiovascular diseases (CVDs) prevalence continues to increase, and it is still the number one killer so far. In 2002, nearly 17 million deaths all over the world were attributable to CVDs, which accounted for almost 30% of the total deaths. Despite treatment with percutaneous coronary intervention (PCI) and many other conventional medicines, CVDs patients are still confronted with certain risk of recurrent acute cardiovascular events, readmission to the hospital, and unfavorable quality of life. In recent years, more and more clinicians have successfully applied complementary/alternative medicine (CAM) in CVDs prevention and treatment based on standardized conventional therapy. Nevertheless, the role of CAM in CVDs still needs more clinical evidence and definite mechanism of actions. 

In this issue, a collection of several original research articles and reviews are presented that address the clinical application and the mechanism of action of CAM in the treatment of CVDs. These works were submitted by researchers from different parts of the world, including China, Japan, South Korea, Australia, and Sweden. In these studies, the effectiveness of Chinese medicine and some other alternative therapeutic methods in improving symptoms was demonstrated in patients with hypertension, chronic stable coronary artery disease, chronic heart failure, and so forth. Specifically, the use of Chinese herbal medicines was reviewed for the prevention of in-stent coronary restenosis after PCI. The study of Tanshinone IIA, a diterpene quinine extracted from the root of salvia miltiorrhiza, a Chinese traditional herb, was presented as a promising cardioprotective agent. The positive effect of Chinese food and herbal medicines in improving certain moderate dyslipidemias was described. The usefulness of Xuezhikang, an extract from Red Yeast Rice, was reviewed in the treatment of coronary heart disease complicated by dyslipidemia. A pharmacological and mechanistic study showed Naoxintong's effect on cytochrome P450 2C19. Further, one study showed the effect of berberine on improving insulin sensitivity by inhibiting fat store and adjusting adipokines profile in human preadipocytes and metabolic syndrome patients. 

In the authors' opinion, the clinical research of Chinese medicine and other CAMs for CVDs still faces some major challenges. Issues such as overall quality of medical service and the unmet medical needs in the contemporary society are common to these medicines. A general guideline is required for practicing Chinese medicine and other CAMs, which should be developed based on solid evidence from well-designed and well-executed clinical studies. Such is the direction that the research of Chinese Medicine and other CAM should follow.

*Ke-ji Chen* *Ke-ji Chen* *Ka Kit Hui* *Ka Kit Hui* *Myeong Soo Lee* *Myeong Soo Lee* *Hao Xu* *Hao Xu*

